# High throughput phenotyping of cross-sectional morphology to assess stalk lodging resistance

**DOI:** 10.1186/s13007-021-00833-3

**Published:** 2022-01-04

**Authors:** Yusuf A. Oduntan, Christopher J. Stubbs, Daniel J. Robertson

**Affiliations:** 1 Department of Mechanical Engineering, University of Idaho, Moscow, ID 83844 USA; 2 School of Computer Sciences and Engineering, Fairleigh Dickinson University, Teaneck, NJ 07666 USA

**Keywords:** Analysis, Cross section, Feature extraction, Finite element, Imaging, Lodging, Morphology, Phenotyping, Stalk, Strength

## Abstract

**Background:**

Stalk lodging (mechanical failure of plant stems during windstorms) leads to global yield losses in cereal crops estimated to range from 5% to 25% annually. The cross-sectional morphology of plant stalks is a key determinant of stalk lodging resistance. However, previously developed techniques for quantifying cross-sectional morphology of plant stalks are relatively low-throughput, expensive and often require specialized equipment and expertise. There is need for a simple and cost-effective technique to quantify plant traits related to stalk lodging resistance in a high-throughput manner.

**Results:**

A new phenotyping methodology was developed and applied to a range of plant samples including, maize (*Zea mays*), sorghum (*Sorghum bicolor*), wheat (*Triticum aestivum*), poison hemlock (*Conium maculatum*), and Arabidopsis (Arabis thaliana). The major diameter, minor diameter, rind thickness and number of vascular bundles were quantified for each of these plant types. Linear correlation analyses demonstrated strong agreement between the newly developed method and more time-consuming manual techniques (R^2^ > 0.9). In addition, the new method was used to generate several specimen-specific finite element models of plant stalks. All the models compiled without issue and were successfully imported into finite element software for analysis. All the models demonstrated reasonable and stable solutions when subjected to realistic applied loads.

**Conclusions:**

A rapid, low-cost, and user-friendly phenotyping methodology was developed to quantify two-dimensional plant cross-sections. The methodology offers reduced sample preparation time and cost as compared to previously developed techniques. The new methodology employs a stereoscope and a semi-automated image processing algorithm. The algorithm can be used to produce specimen-specific, dimensionally accurate computational models (including finite element models) of plant stalks.

**Supplementary Information:**

The online version contains supplementary material available at 10.1186/s13007-021-00833-3.

## Background

Stalk lodging (structural failure/breakage of plant stalks) is a multi-billion dollar a year problem experienced by many crops [1, 2]. Stalk lodging occurs when bending moments along the stalk induced by a combination of external loads (e.g., rain, wind) and self-loads (e.g., plant weight) exceed the stalk’s bending strength [3–6]. This leads to stalk failure, typically in form of buckling [7]. Several vital crop species that are key to maintaining global food and energy security are particularly prone to stalk lodging. For example, maize, rice, and wheat collectively account for two thirds of human caloric intake and are also used for biofuel production [8]. All three of these crops are severely affected by stalk lodging. Global annual yield losses due to stalk lodging are estimated to range from 5 to 25% annually [9, 10]. The cross-sectional morphology of stalks and in particular, morphological measurements including rind thickness and diameter are key determinants of stalk bending strength and lodging resistance [11–14]. However, current methods of measuring cross-sectional morphology of plant stalks are time consuming, and often require specialized equipment and expertise [15].

Phenotyping of cross-sectional morphology is typically accomplished via either: histologically prepared samples with microscopy imaging [16, 17], hand cut sections with flatbed scanner imaging [18] or X-ray computed tomography imaging [19–23]. Histology and microscopy-based phenotyping typically requires at least a day for sample preparation (i.e., sample fixation, staining and sectioning). Hand sectioning and flatbed scanning is significantly faster and cheaper but has limited resolution and is not well suited to plants that are dry (not hydrated), hollow or have smaller diameters. X-ray computed tomography methods require expensive imaging equipment and typically require hours to obtain an image depending on the desired resolution. Additionally, X-ray imaging typically performs poorly on hydrated plant samples unless contrast enhancing agents are employed.

This study presents an imaging and digitization methodology that offers reduced sample preparation time as compared to typical histology methods, while producing higher imaging resolutions than flatbed scanning. The method is relatively inexpensive as compared to X-ray computed tomography and does not require the user to possess specialized imaging expertise. The methodology enables measurement of stalk diameter, rind thickness and vascular bundle counts of two-dimensional plant cross-sections. In addition, users can easily export the cross-sectional geometry into third party software for further analysis (e.g. finite element modeling). The sample preparation protocol includes a simple sectioning and staining procedure followed by imaging under a stereo microscope. Digital images from the microscope are then read into an image processing algorithm to produce measurements of stalk cross-sectional geometry. The image processing algorithm produces a Python script that can subsequently be executed to generate specimen-specific finite element models of plant stalks. Finite element models are an advanced computational tool used by structural engineers to analyze the strength and deflection of loaded members. Finite element models of plants can be used to analyze stalk strength and lodging resistance and produce understanding that cannot typically be obtained via field or lab-based experiments [11].

One challenge to creating finite element models of plant stems is creating an accurate digital representation of plant geometry/morphology. Creating an accurate model geometry is important as both physical experiments [24] and in silico studies [4] have demonstrated that even small morphological features of plant stalks have a strong influence on stalk strength and lodging resistance. Previous finite element models of plant stalks have utilized X-ray computed tomography scans [4, 5, 11, 21, 23], caliper measurements [6, 25] or simple approximations [26] to create model geometry. Computed tomography scans produce the most accurate geometric representation for finite element analysis. In particular, computed tomography scans accurately quantify cross-sectional features along the entire length of plant stalks including rind, pith and vascular bundle boundaries. Caliper measurements are not as accurate as computed tomography scans. It is very challenging to accurately quantify the rind-pith boundary with calipers and they are not well suited to quantifying or digitizing vascular bundle boundaries [15]. Simple approximations of geometry (e.g., assuming a circular cross-section) are the least accurate representation of plant geometry. The newly developed cross-sectional phenotyping methodology presented in this paper is not as accurate as computed tomography scans. However, it is significantly faster and cheaper than computed tomography and provides a more accurate representation of cross-sectional geometry as compared to caliper measurements.

To evaluate the newly developed methodology, obtained measurements of rind thickness, diameter and vascular bundle count were compared to traditional manual measurements. In addition, dimensionally accurate, specimen-specific finite element models of plant stalks were created to demonstrate the utility of the methodology.

## Results

**Fig. 1:**
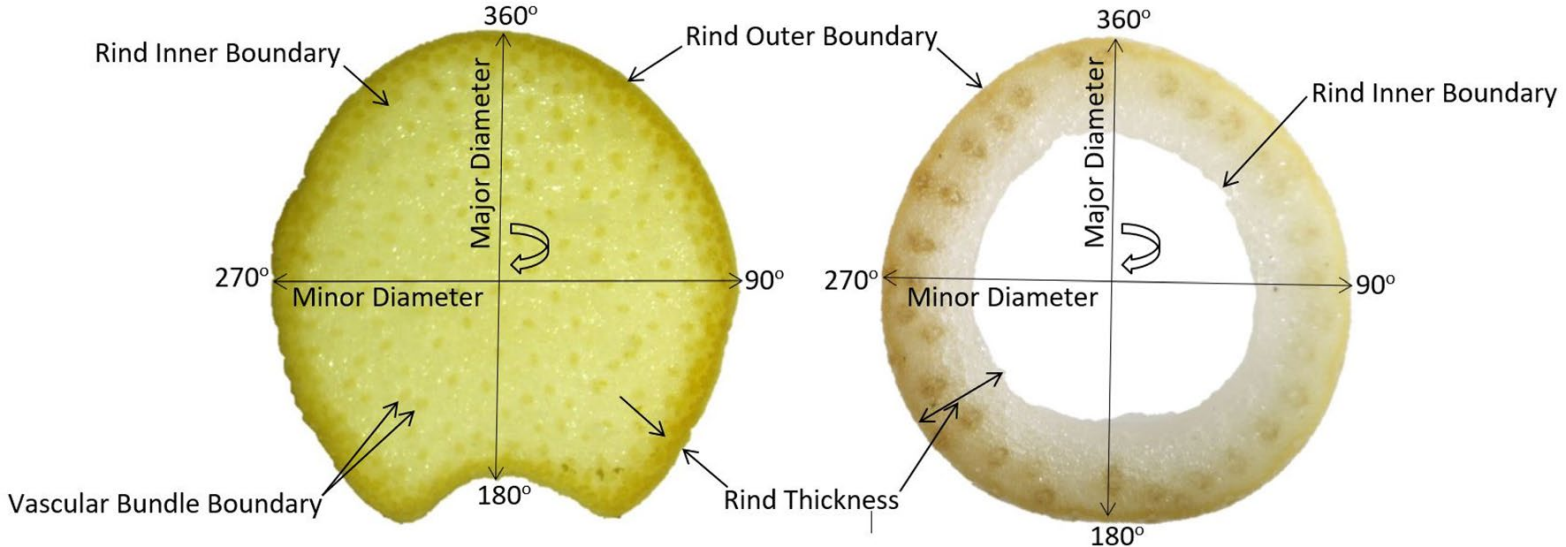
Cross-sectional phenotypes commonly associated with stalk strength and stalk lodging resistance. Maize (left), and wheat (right)

Dried plant stalks were used as samples for this study. Plants analyzed included maize (Zea mays), sorghum (Sorghum bicolor), wheat (Triticum aestivum), poison hemlock (Conium maculatum), and Arabidopsis (Arabis thaliana). Figure 1 depicts common cross-sectional phenotypes associated with stalk strength and stalk lodging resistance that were quantified for each of the samples in this study. The phenotyping methodology used to quantify these phenotypes can be separated into three distinct stages: (1) sample preparation, (2) imaging, and (3) feature extraction. The following paragraphs present results obtained from each of these stages. Standard operating procedures and protocols for sample preparation, imaging and feature extraction of each plant type are provided as Additional file 1.

### Stage 1: sample preparation

Several sectioning methods were investigated to determine which would produce the highest quality sections. We investigated the use of razor blades, waterjet cutters, and power saws with both abrasive and toothed blades. Ultimately a 1/4 horsepower, variable-speed, direct-drive trim saw machine produced by Reentel International Inc. (Westmont, IL) was found to produce the highest quality sections of maize, sorghum, and poison hemlock. In general, results revealed the optimal blade type for sectioning was a toothless abrasive type blade. In particular, a 6-inch, 120-grit thin notched diamond saw blade (also purchased from Reentel International Inc) produced acceptable plant sections. The blade was operated at adjustable speeds to prevent burning of plant sections. Plant stalks were typically cut at speeds between 1300–2300 rpm depending on the hardness of the plant stalk’s rind. The saw fence (Hi-Tech Diamond 70–300) and saw vise (Hi-Tech Diamond 22–331) ensured the cutting of parallel and thin 2–4 mm sections. A razor blade (Van Der Hagen Stainless Razor Blades) was the best tool for cutting quality sections of the wheat and Arabidopsis plants. To prevent crushing, the wheat and Arabidopsis plants were hydrated before cutting sections. To produce parallel sections with even imaging surfaces, it was essential to repeatedly cut adjacent sections with the razor blade by chopping down in a single smooth motion without any back-and-forth sawing motion.

A differential staining technique (i.e., staining and counter staining) produced the required contrast between plant structures. In particular, an Alcian Blue-Safranin O sequence [27] was selected as the best universal staining method for all plant species as it ensured clear delineation of the boundary between the rind and pith tissues as well as identification of vascular bundles within the pith and the rind.

### Stage 2: imaging

**Fig. 2:**
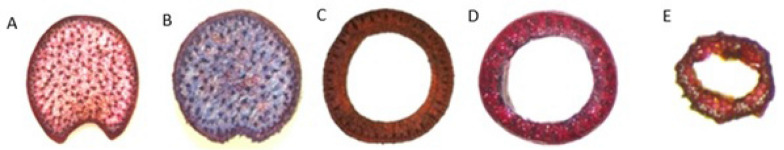
Digital images of **A** maize, **B** sorghum, **C** poison hemlock, **D** wheat and **E** arabidopsis

Images were acquired using a stereo microscope camera (AmScope LED Trinocular Zoom Stereo Microscope with 18MP digital camera) [28] and lens (0.5× and 2.0× Barlow lenses). This setup produced sharp-quality images that enabled identification of plant structures as shown in Fig. 2. A spatial calibration factor for each image was obtained using the associated microscope software. Digital cross-sectional images were automatically batch saved in folders.

### Stage 3: feature extraction

**Fig. 3:**
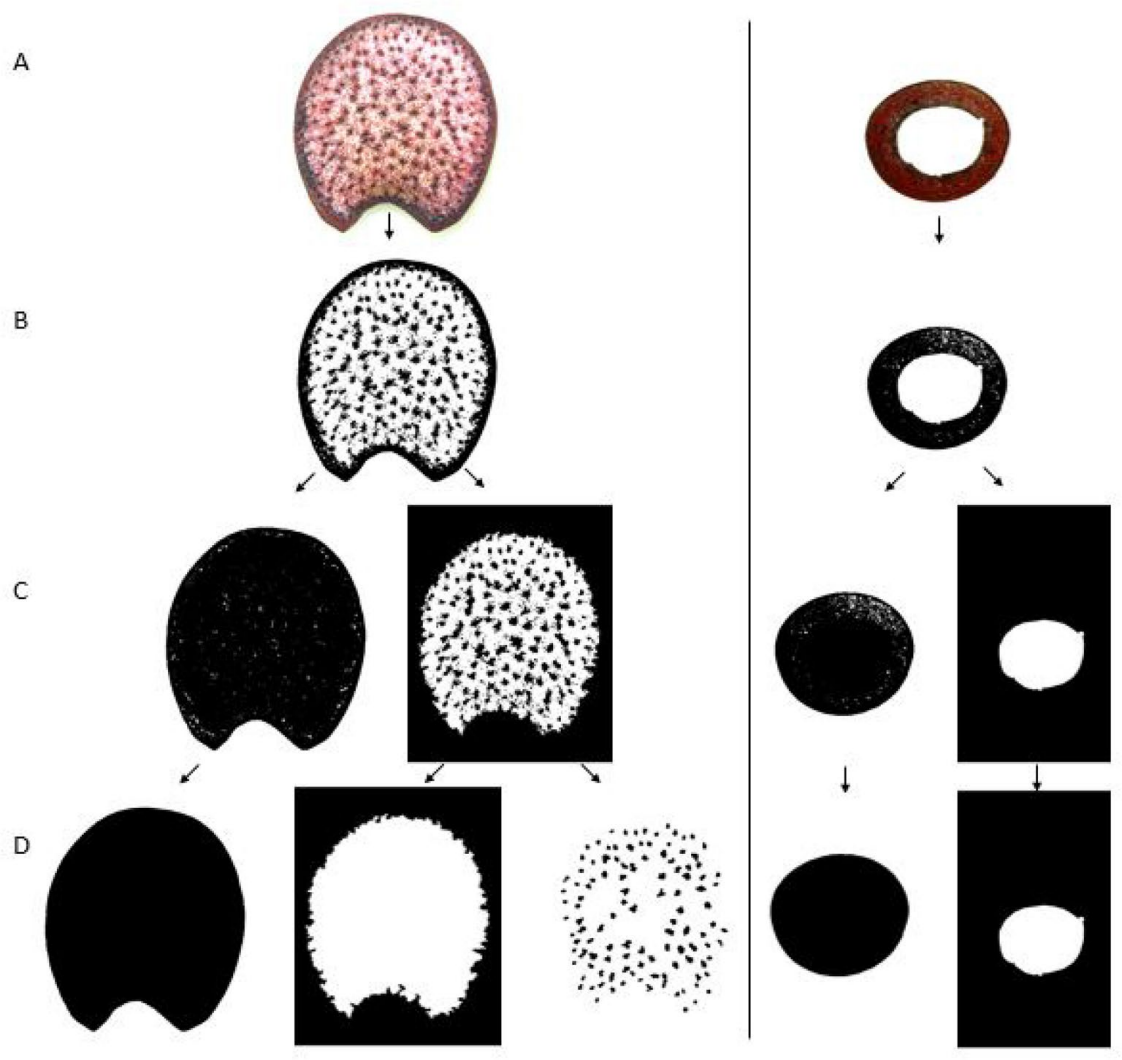
**A** Raw (RGB) images, **B** thresholded (binarized) images, **C** segmented images, and **D** Smoothed images of maize and wheat stalk sections

Feature extraction was accomplished using a custom algorithm developed in the MATLAB (MATLAB R2019) software environment [29]. The algorithm quantified cross-sectional phenotypes using several built-in image processing functions. The first step in this process was to convert the raw red, green, and blue digital image (see Fig. 3A) to a binarized image. In the binarized image highly lignified tissues appear as black pixels and other tissues appear as white pixels as shown in Fig. 3B. The binary images were subsequently segmented as shown in Fig. 3C. In particular, the “whole cross-sectional area” and the “pith area” or “hollow area” of the cross-section were segmented into distinct images. This segmentation generally revealed hidden unwanted pixel clusters in the whole cross-sectional area image. These unwanted pixel clusters (small white elements in the rind and small black elements in the pith) can be seen in Fig. 3C. These pixel clusters were cleared using automated smoothing operations (see Fig. 3D).

**Fig. 4:**
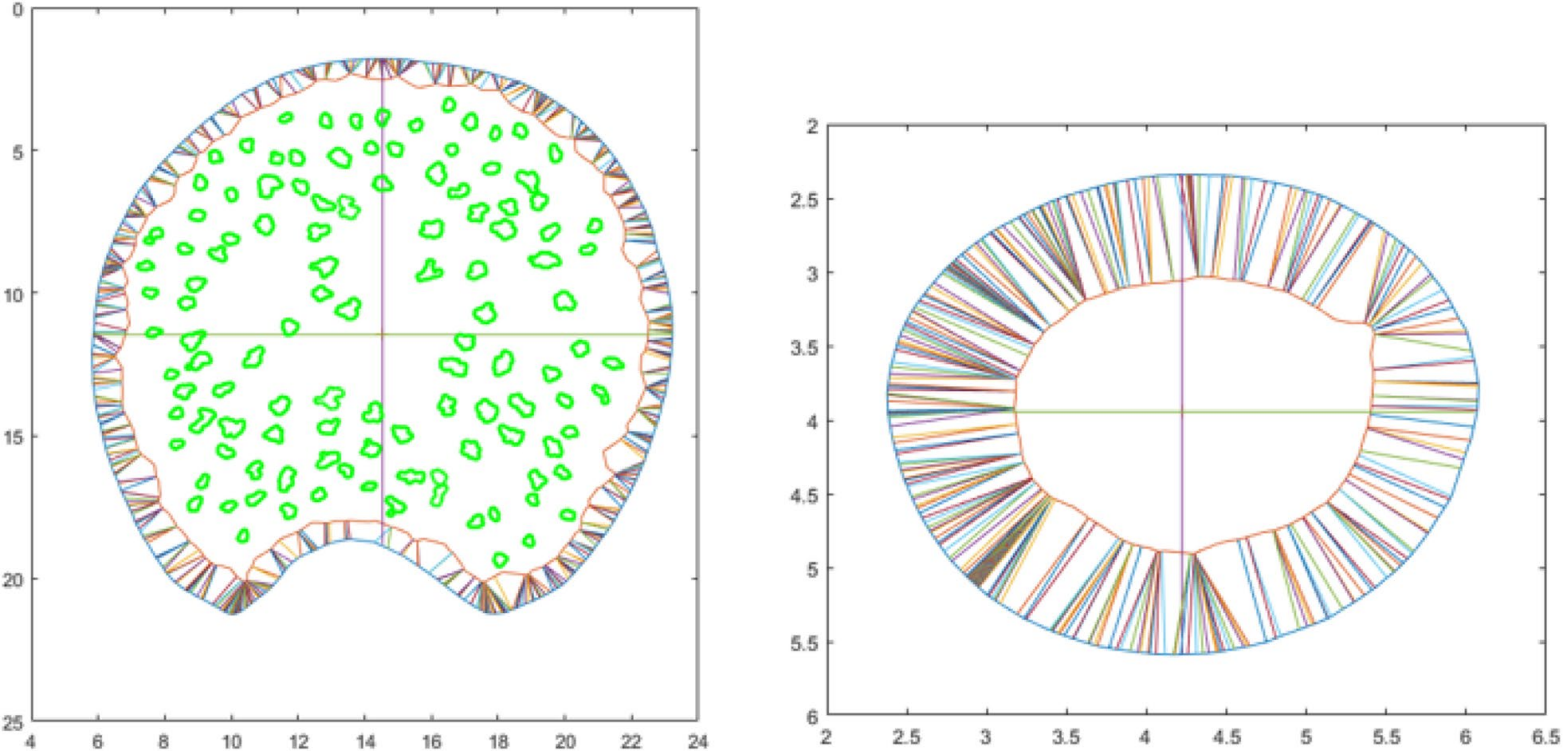
Maize section (left) and wheat section (right) showing extracted rind boundaries, major and minor diameter lines, rind thickness measurement lines and maize vascular bundle boundaries (left)

**Fig. 5:**
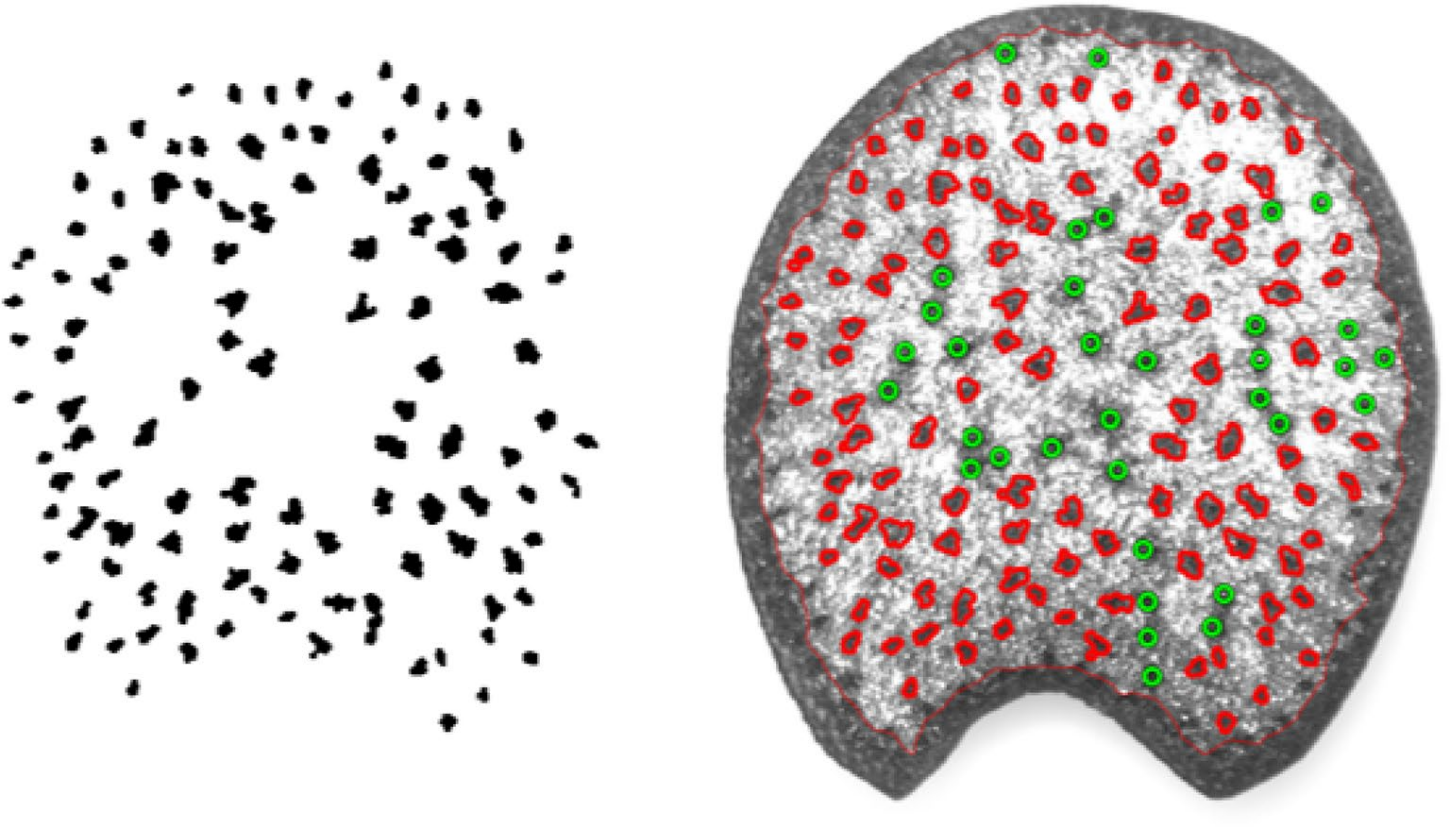
A maize section’s automatically identified vascular bundles (left). A grayscale image of the maize section with automatically identified vascular bundle boundaries shown in red and manually selected vascular bundles shown as green circles (right)

Once the images had been segmented, they were analyzed to determine the major and minor diameter of the cross-section as well as the rind and vascular bundle boundaries (see Fig. 4). The rind thickness is determined by plotting the shortest possible line segment between each point of the outer rind boundary and any point of the inner rind boundary as shown in Fig. 4. The average length of all the line segments was calculated as the rind thickness. The algorithm then identifies the vascular bundles and their boundaries. Next, the vascular bundle boundaries are overlaid on top of the grayscale image and the user can manually select vascular bundles that the algorithm may have missed (Fig. 5).

**Fig. 6:**
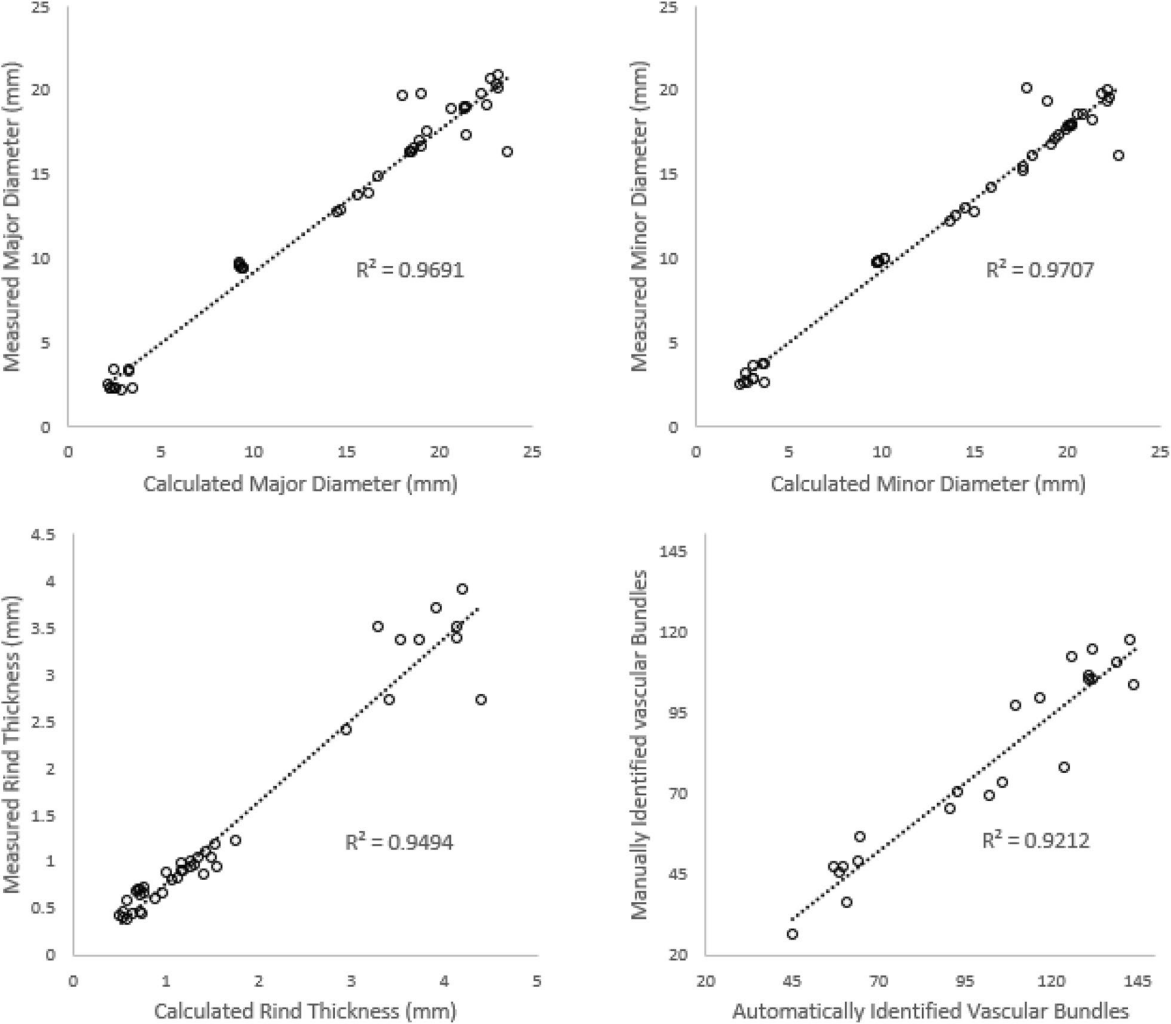
Image analysis algorithm vs manual measurements of wheat, sorghum, maize, and poison hemlock stalks

Each cross-sectional phenotype obtained by the newly developed image-analysis algorithm was compared to traditional manual measurements. As can be seen in Fig. 6, results from the image-analysis algorithm were in good agreement with manual measurements. In particular, linear-correlations analysis demonstrated R^2^ values of 0.9691, 0.9707, 0.9494 and 0.9212 for the major diameter, minor diameter, rind thickness, and vascular bundle count, respectively.

### Exporting extracted plant phenotypes to third party software

Specimen-specific cross-sectional geometries (i.e., vascular bundle boundaries as well as outer and inner rind boundaries) were exported into Abaqus [30] which is a third-party finite element analysis software. Dimensionally accurate finite element models generated in third-party software like Abaqus can be used to further assess stalk strength and lodging resistance [11]. We created finite element models with specimen-specific geometries and subjected them to transverse compression and bending loads. The stiffness response of the stalk’s internodes and locations of maximum stress along the stalk’s internodes were determined. Details about each of the models are given below.

**Fig. 7:**
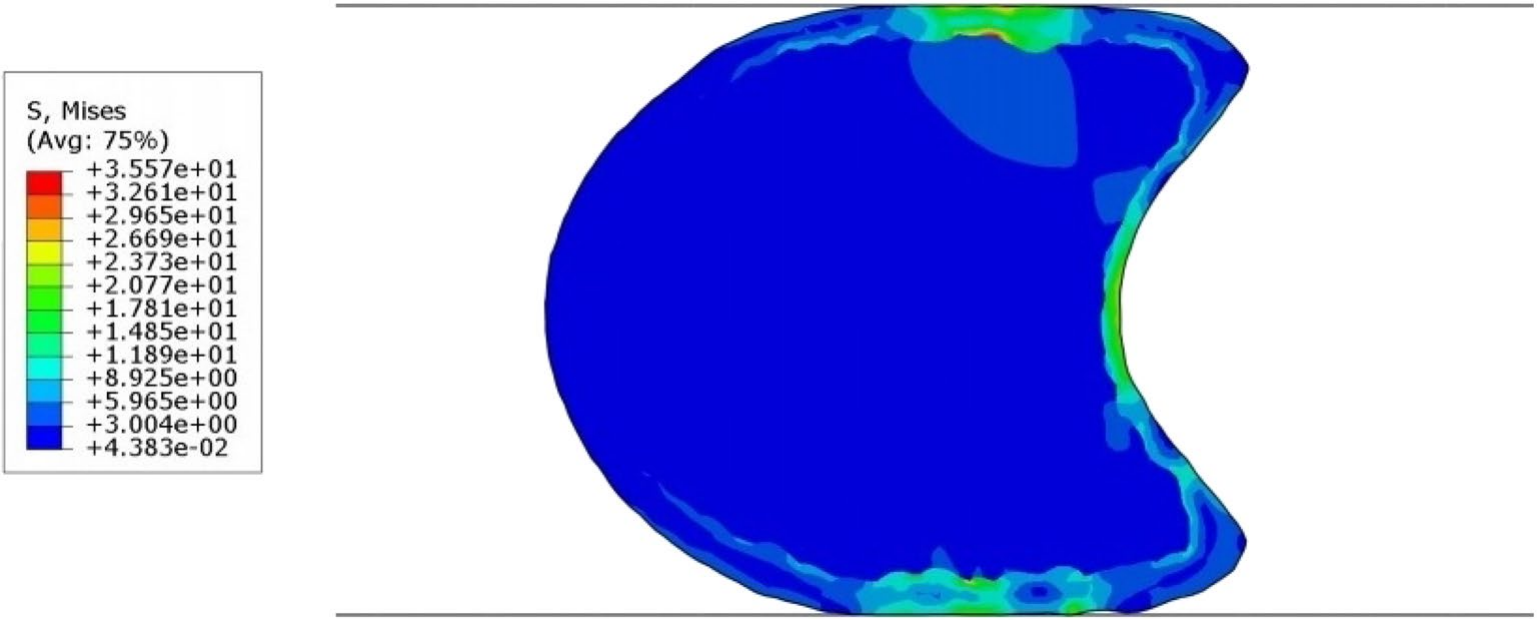
Deformed model of maize stalk loaded in transverse compression, showing Von Mises stress (MPa) experienced (See Mesh in Fig. 12)

Five finite element models of five internodes of a maize stalk were analyzed in transverse compression. The reaction force of maize internodes subjected to compressive loading ranged between 15 and 18 N. For the bottom three internodes, the model’s maximum mechanical stresses under transverse compression were concentrated near the loading platens. However, the top two internodes exhibited a different stress contour, as was expected based on their unique geometry (i.e., the presence of longitudinal groove in the stalk). In particular, for the top two internodes, high mechanical stresses were also distributed along the edges of sample as shown in Fig. 7.

**Fig. 8:**
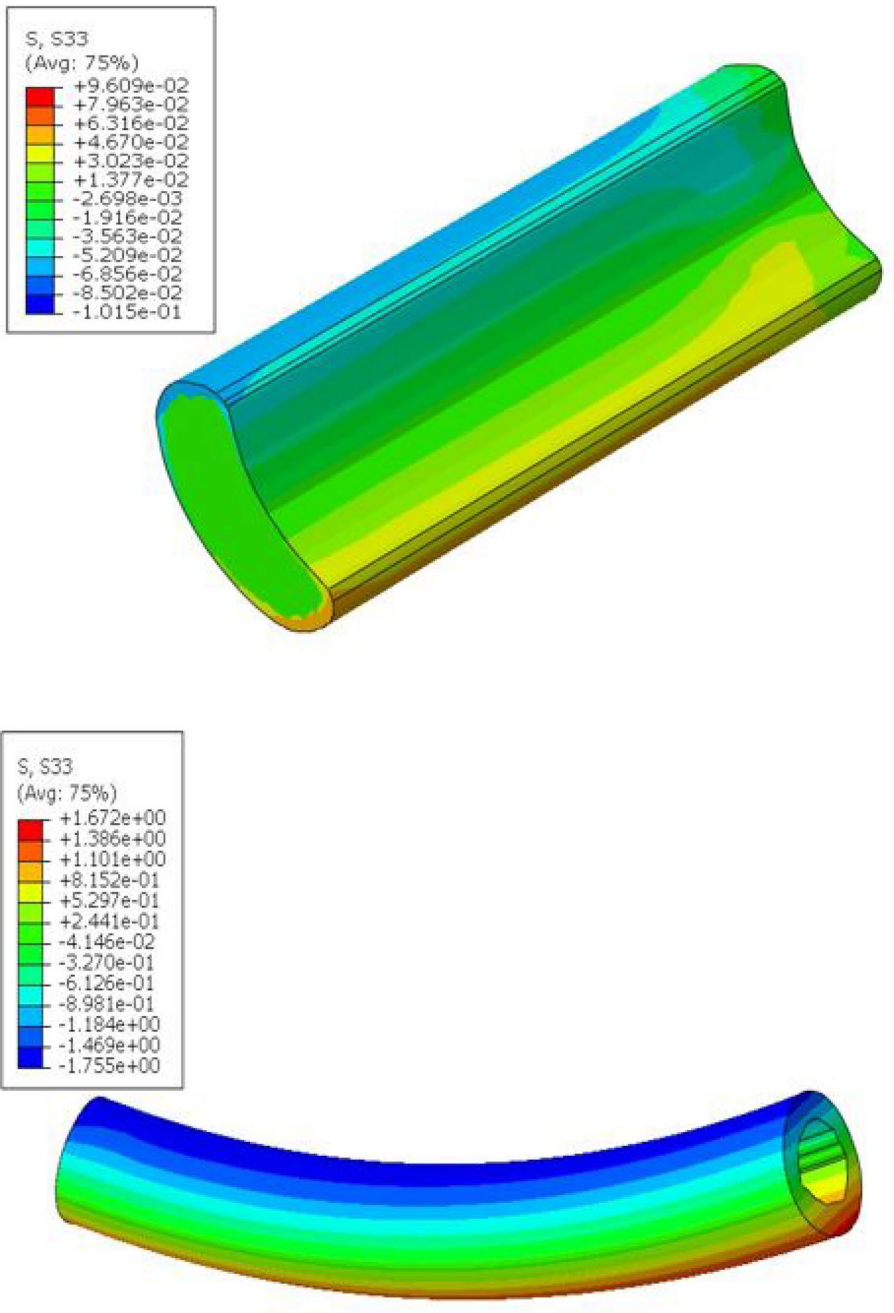
Deformed model of maize (top) and wheat (bottom) stalk internodes under bending showing principal stress (MPa) contours along stalk (See mesh in Fig. 13)

Two additional finite element models (one of a wheat stalk internode and one of a maize stalk internode) were created and analyzed in bending. The maximum mechanical stress experienced by the wheat stalk internode was 1.672 MPa under a bending moment of 1Nm. The maize stalk internode experienced maximum stress of 96.09 kPa under a bending moment 1Nm. As can be seen in Fig. 8 both models depict similar stress contours along the length of the internode. As expected, the top and bottom surfaces of the internode’s loading axis experienced the maximum mechanical stresses.

## Discussion

Cross-sectional morphology is a strong determinant of stalk bending strength and stalk lodging resistance [4, 14]. A simple and cost-effective method for quantifying cross-sectional morphology has been developed. This methodology can be applied to hollow and pith-filled stalks to quantify rind thickness, major and minor diameter, and number of vascular bundles. The method can also be used to export the obtained cross-sectional morphology into third-party software for further analysis. The methodology requires the use of a trim saw or razor blade for sectioning, a relatively inexpensive stereo microscope for imaging, and an opensource image analysis algorithm. The total cost of equipment required to implement this methodology is approximately $2250. Researchers can use this new methodology to produce specimen-specific finite element models of plant stalks in under 24 h. Prior to developing this phenotyping methodology, it typically took our lab over three weeks to develop a specimen-specific finite model of a plant stalk.

The ability to create specimen-specific finite element models of plant stalks in under a day is expected to enable more studies in plant biomechanics. Some plant studies have used finite element analysis [5, 11, 12, 21–23, 31] previously. However, many studies have been impeded by the high cost of creating specimen-specific finite element models of plants. Once developed specimen specific finite element models of plants can be modified and evaluated in minutes as opposed to field experiments which can take a year or more. Using finite element analysis techniques, experiments can be repeated under precise loading conditions that are difficult to replicate in the lab due to the complex, irregular, and unique geometries of plant stalks. Furthermore, finite element models enable precise and independent control of each model parameter (i.e., rind thickness, diameter, vascular bundle count etc.) a feat that is nearly impossible in field or lab-based experiments. These unique features of finite element models enable application of engineering techniques that can be used to better decipher the role of morphology in stalk lodging resistance (e.g., topology optimization and sensitivity analysis).

For example, analysis of failure patterns from naturally lodged plant stems and laboratory-based experiments indicate that lodged plant stems typically fail in one of three ways: compressive tissue failure at the outer fiber, transverse buckling, or longitudinal splitting [22, 32, 33]. Each of these failure modes is strongly influenced by the morphological properties of the stalk [4, 11, 12]. In particular, rind thickness and diameter are known to be key determinants of stalk bending strength and lodging resistance [11–14]. Previous finite element studies of maize [4] and wheat stalks [34] have been conducted to investigate stalk lodging and transverse buckling of plant stems. Other studies have assessed bending stresses [5], the effect of stress concentrations [11], and material properties [21, 23] in plant stems. The newly developed phenotyping methodology presented in the current study will enable similar studies to be conducted in the future at reduced time scales and reduced cost.

In developing this phenotyping methodology, several sectioning, staining, and imaging procedures were investigated. We found that the methodology presented in the results sections provided the optimal balance of cost and quality and required little to no specialized expertise or training. Standard operating procedures and protocols for using this methodology for pith filled and hollow plant stems are provided as Additional file 1 to enable other researchers to easily apply the new methodology. The code for the image-processing algorithm is also provided as Additional file 2. Sample images and instructions are provided as Additional file 3.

**Fig. 9:**
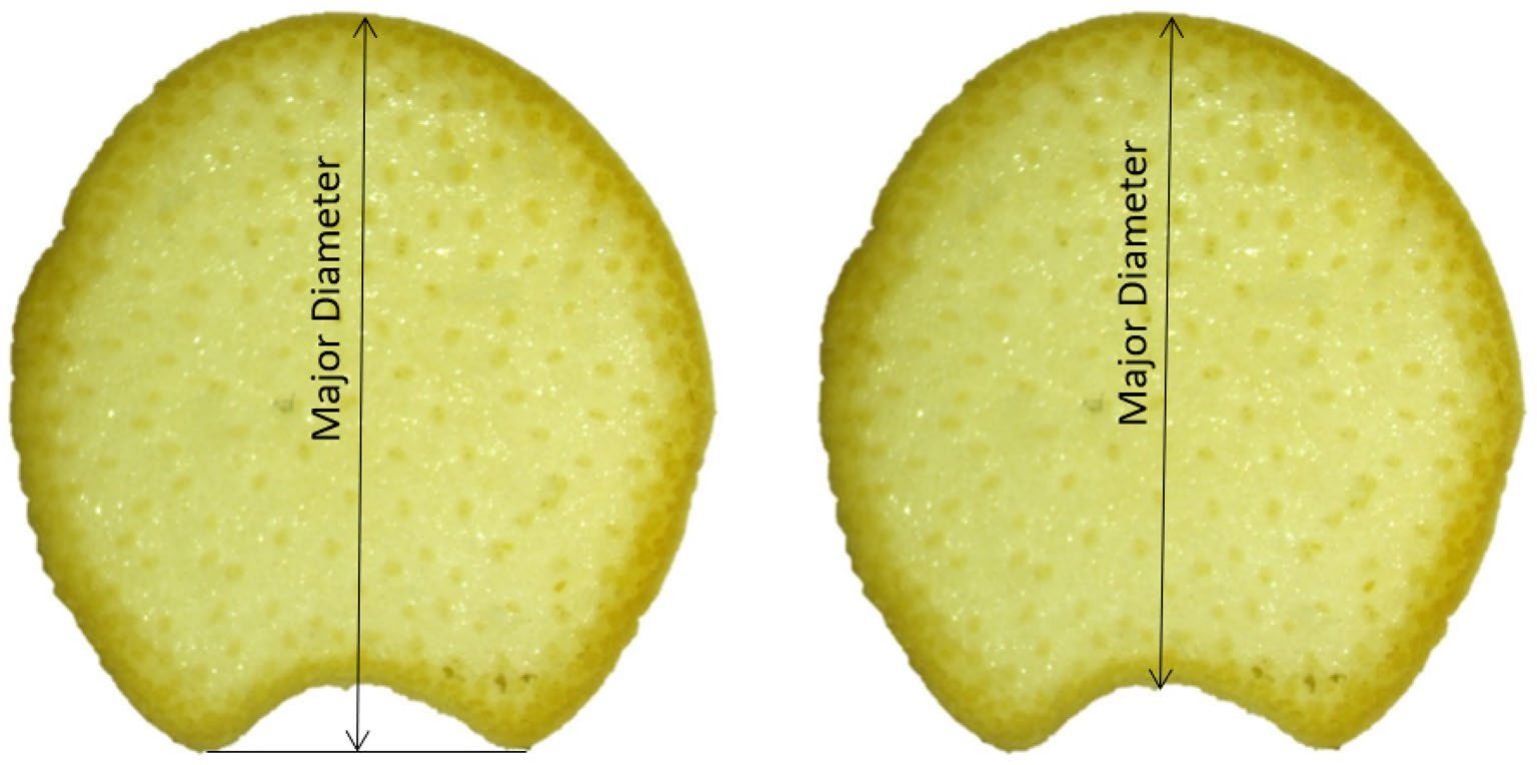
Major diameter manual measurement (left) and algorithm calculated measurement (right)

In general, the newly developed phenotyping methodology is in good agreement with manual measurements. In particular, linear correlations analyses demonstrated R^2^ values greater than 0.9 for measurements of major diameter, minor diameter, rind thickness and vascular bundle count. However, some differences between manual measurements and the newly developed methodology were observed. In particular, the newly developed method calculates rind thickness as the average length of all minimum possible line segments between the inner and outer rind boundaries. However, manual measurements of rind thickness are typically acquired at just a few points and are then averaged. Thus, manual measurements are dependent on the selected points and are also affected by how hard the caliper jaws squeeze the rind tissue. In addition, the parallel jaw faces of a caliper necessitate quantifying the major diameter as shown in the left panel of Fig. 9, whereas we quantified the major diameter as shown in the right panel of Fig. 9. Although major diameter results presented in this paper were calculated as shown on the right side of Fig. 9, the MATLAB code can also be used to calculate the major diameter as shown on the left side of Fig. 9 by uncommenting a section of a the code.

### Limitations

Dry and mature stalks were used in this study. Dry stalks were chosen as the authors were interested in late season stalk lodging that occurs after the plant has reached full maturity and dried down in the field. Dried stalks were also used to prevent spoilage and eliminate discrepancy from stalk moisture content variation. Further development of this methodology could involve the investigation of moisture content and turgor pressure on stalk morphology.

## Methods

### Sample preparation

#### Sectioning

We aimed to create a sample preparation protocol that was cost effective and produced high quality plant sections. All plants included in this study exhibited transversely isotropic morphologies with vascular bundle fibers oriented in the longitudinal direction (e.g., see scanning electron microscopy [22] and computed tomography [12] images). The composite/transversely isotropic nature of plant stalks makes them especially difficult to cut without damaging the cross-sectional morphology of interest. Lignified vascular bundles are particularly difficult to cut without tearing or crushing of adjacent tissue structures. Common problems encountered when trying to section plant stalks include: tearing of vascular bundles, crushing of adjacent tissue structures, oblique faces of plant sections, and uneven/rough imaging surface. Table 1 shows the tools and blade types that were investigated in this study. Each of these tools/blade types was evaluated based on the cost of the tool and the quality of the resulting plant section.

**Table 1:** Evaluation of cutting machines for plant sectioning

Problems	Chop saw	Angle grinder			Water jet	Trim saw
		Coarse blade	Fine blade	Tile blade		
Burnt/discolored imaging surface		X				
Ripped or torn rind	X	X	X			
Pulled vascular bundles	X	X	X	X		
Uneven imaging surface	X	X	X	X	X	

The trim saw (Hi-Tech Diamond 6″ Trim Saw) [35] was set up with a Hi-tech Diamond Silver Thin Notched Saw Blade, which rotated between 800 and 3400 rpm, a saw fence and a saw vise [35]. Sections of maize, sorghum and poison hemlock plants were cut with the smooth, unpainted, and toothless saw blade at speeds typically between 1200 and 2000 rpm. The plant was held firmly with the saw vise and pushed through the rotating blade to cut sections. Sections were cut to required thickness by adjusting the saw fence accordingly. To avoid burnt section surfaces, it was essential to ensure pushing the samples through the rotating blade in a quick manner.

The water jet cutter (Omax Model 55100) [36] was operated on the fiberglass cutting setting. Sections produced with the water jet cutter were unburnt and undamaged but required relatively long time periods to cut and it created an uneven/oblique imaging surface. Sections with uneven surfaces subsequently resulted in poor image quality and unacceptable feature extraction results.

The angle grinder (Metabo WEV15-125) [37] and chop saw (Makita 2414DB Cut-Off Saw) [38] were mounted on a pivoted stand that had a built-in clamp to hold plant stalks firmly while cutting. The angle grinder was operated at a blade speed of 3000 rpm. The rotating blade was pushed down to cut through the clamped plant stalk. Plant stalk sections were cut with the angle grinder using three blade types:Piranha Tile Premium Diamond Blade (Tile blade) [39], T1 Premium Thin Cut-off Wheel (Fine blade) [40] and Resin-bonded Aluminum Oxide Flap Disc (Coarse blade) [41]. The tile blade produced the best results with the angle grinder as it produced unburnt sections and prevented ripped rinds. However, the tile blade pulled the vascular bundles embedded in the pith which caused poor image quality. The chop saw was set up with a 14″ abrasive cut off wheel which rotated at 3800 rpm blade speed. The chop saw produced damaged and unacceptable plant sections.

Sections of wheat and Arabidopsis plant stalks were cut using a razor blade. However, the wheat and Arabidopsis samples had to be hydrated before being sectioned by immersing them in distilled water. This reduced friction and enabled easier cutting of thin sections. The plant was held firmly with the thumb and index fingers in one hand and cut with the other. To avoid cutting of oblique sections the blade was held perpendicular to the sample and care was taken to ensure the blade was not bent while cutting. It was important to avoid any sawing motions while cutting sections and instead choping down in one single smooth motion. This prevented ripped rind and sample damage.

#### Staining

Plant sections were stained to improve contrast between plant structures thereby enhancing image analysis and feature extraction. Both simple and differential staining techniques were investigated. These included Alcian Blue, Safranin O, Toluidine Blue, and Alcian Blue-Safranin O staining techniques. The staining protocol for all sections included hydrating sections in distilled water, staining sections by submersion in a solution, rinsing in distilled water to remove excess staining solution followed by dehydrating the section in alcohol.

**Fig. 10:**
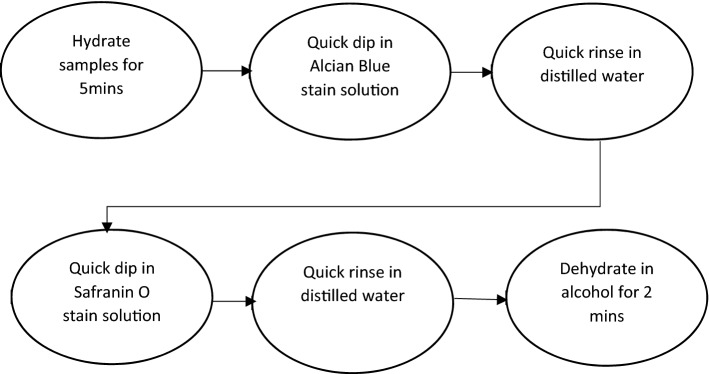
Alcian Blue-Safranin O staining sequence

Differential staining of sections was achieved by following the Alcian Blue-Safranin O staining sequence (Fig. 10) [27].

### Imaging

We sought to employ an imaging setup with a wide field of view to enable the entire cross-section to be captured in a single picture. Adjustable magnification was desired to enable imaging of plant sections with different diameters. An AmScope LED Trinocular Zoom Stereo Microscope along with its 0.5× and 2.0× Barlow lenses and an 18MP digital camera [28] were selected as the best imaging option. The microscope had an objective lens with 0.7 to 4.5 magnification power when used along with its 0.5× and 2.0× Barlow lenses. The 0.5× lens enabled a large working distance for easy adjustment of sections and a wider field of view for the large cross-section of maize, sorghum, and poison hemlock sections. The 2× lens enabled extra zoom to capture smaller sized wheat and Arabidopsis sections.

The first step of the imaging process was to ensure the sharpest (i.e., focused) image of the stained section was viewed on the Amscope camera software interface (Toupview) [28] before capture. The next step was to calibrate the microscope to ensure accurate spatial conversion of section sizes. This was done using a scale rule to obtain the number of pixels equal to 1 mm. After calibration, section images were captured using the digital camera in combination with the AmScope software interface. Each image was saved in tagged image file (tif) format with a dimension of 4912 × 3684 pixels.

### Feature extraction

Feature extraction from digitized plant images consists of four parts, namely: pre-processing, segmentation, smoothing, and quantification. The MATLAB (MATLAB R2019a) [29] computer programming software was used to develop an algorithm to implement each of these steps. The MATLAB code can be found in the provided Additional file 2. The MATLAB feature extraction algorithm was customized to work on both hollow and pith-filled plant types.

Pre-processing consisted of separating the pixels of digital images into groups to aid in distinguishing between different plant structures. To accomplish this the captured Red, Green and Blue (RGB) digital image was first converted to a grayscale image. This conversion reduced the red, green, and blue intensity channels to one intensity level per pixel. Pixels were then grouped according to their pixel intensity values using local adaptive thresholding [42] and Otsu thresholding [43] for pith-filled and hollow plant type respectively. Optimum threshold values were automatically calculated and applied to different regions of the image to aid in identifying plant structures. The image was then binarized to consist of either black or white pixels.

Segmentation involved identifying and separating different plant structures from the binary image. Maize and sorghum binarized sections were segmented into two different areas. The largest area (whole cross-section) in the binary image and the second largest area (the pith). Single material plant types were segmented into their hollow and whole cross-sectional areas. To ensure adequate identification and measurement of plant structures, image smoothing was applied to segmented areas.

Smoothing operations were implemented to remove unwanted details from segmented images. Unwanted details were identified as objects in images with very small area. The fill and erosion operations were used to smooth images [44]. Both processes required creating a new structuring element—an object with a specified radius or area and pixel, which is placed at the center of each object in the image to be smoothed [44]. Any image object smaller than the newly created object automatically picks up the pixel of the newly created object.

Quantification involved the extraction and measurement of identified plant structures. The rind boundary coordinates were used to quantify stalk diameters and rind thickness. The major and minor diameter endpoints were calculated using the rind outer boundary coordinates at 90°, 180°, 270° and 360^o^ as shown in Fig. 1. The rind thickness was calculated as the mean of the shortest distance between each pair of the inner and outer rind boundary coordinates. These diameter and rind thickness measurements were validated in comparison to digital calipers measurements. The major and minor diameter were measured with digital calipers using the same orientation of the acquired image read into the image processing algorithm. The average rind thickness of each section was calculated by taking digital caliper measurements of the rind thickness at 90°, 180°, 270° and 360° as shown in Fig. 1.

In maize and sorghum sections, vascular bundles were quantified using a semi-automatic approach. First, the number of vascular bundles was automatically quantified. Then closely packed, collectively identified vascular bundles were deleted and individually re-selected. At this time any omitted bundles were also selected. The boundary of each vascular bundle was also extracted. These extracted rind and vascular bundle boundaries were imported into third party software for further analysis.

The image processing algorithm was developed to be compatible with microscope images of stained cross-sections of plant stalks. However, with minimal modification the algorithm can also work with other high-resolution and high-quality images. For example, the authors were able to use the algorithm to analyze and extract rind boundaries from a computed tomography image. However, when used in conjunction with low-resolution images obtained via a flatbed scanner, the algorithm was not as effective.

### Exporting extracted plant geometry

**Fig. 11:**
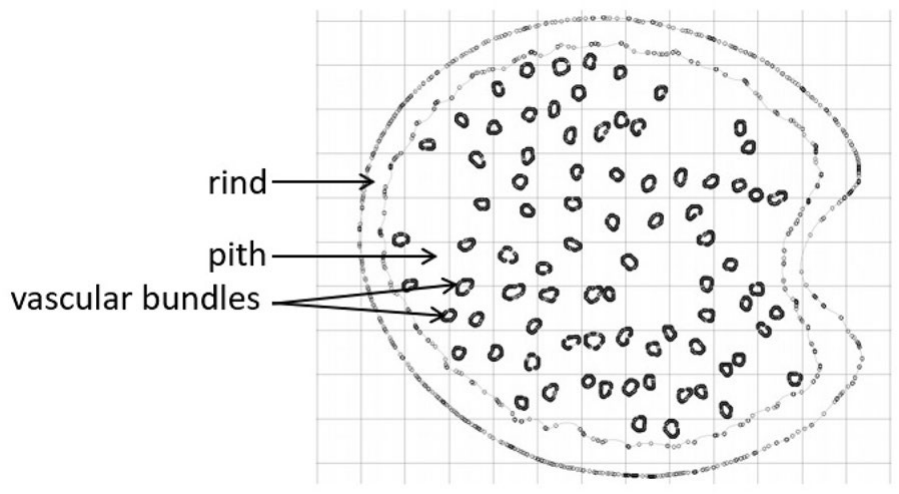
Imported maize stalk specimen-specific cross-sectional geometry

Specimen specific cross-sectional geometries were extracted from digitized plant sections and imported into Abaqus software to create finite element models for analysis. In particular, the pixel location of geometric boundaries were converted to world coordinates in units of millimeters. This unit conversion was done using the MATLAB imREF2D function [29] and the calibration obtained during image acquisition. The MATLAB algorithm then created a Python script file that could be executed in Abaqus to create the specimen specific geometry. In particular, the MATLAB code printed the command statements required to create a model, specify a sheet size, sketch splines, and rename sketches in Abaqus. The MATLAB code also printed the rind and vascular bundle spline coordinates. Once the Python script executed in Abaqus it created a sketch as shown in Fig. 11.

**Fig. 12:**
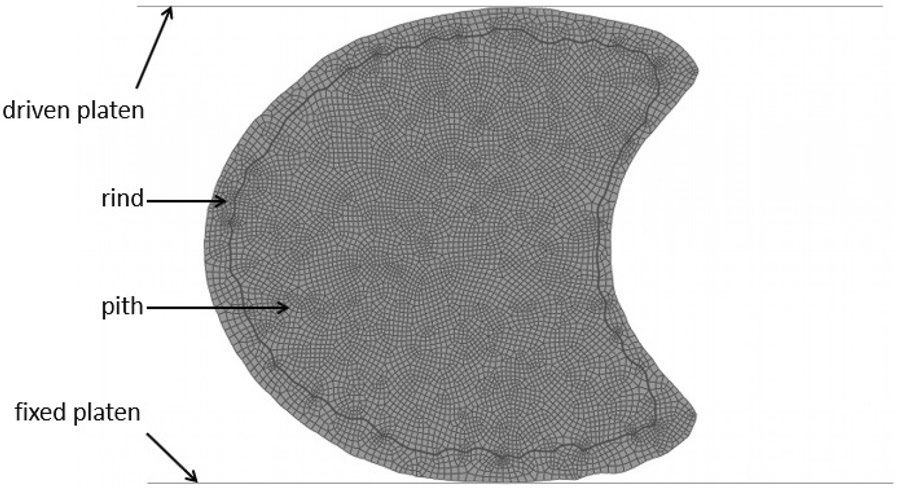
Undeformed two-dimensional model of maize stalk in compression

The specimen-specific geometries imported into Abaqus were used to create two-dimensional finite element models. These models were subjected to transverse compression analyses. Quantifying anisotropic material properties of plant stalks is challenging. However, it is common to use a transversely isotropic material assumption when modeling plant stems [4, 5, 11, 21, 23]. In this study the maize stalk rind and pith materials were modeled as transversely isotropic. The transverse Young’s modulus of the rind and pith materials were set as 850 MPa and 26 MPa, respectively. While the Poisson’s ratio was set as 0.25 [21]. As can be seen in Fig. 12, the geometries were meshed at a global seed size of 0.2 mm with four-node bilinear plane stress reduced integration quadrilateral elements with hourglass control. Two rigid body platens were used to compress the stalk model (see Fig. 12). The top platen was fixed in the rotational and horizontal direction and lowered in the vertical direction until it had compressed the stalk cross-section by 1 mm. The bottom platen was fixed in all degrees of freedom.

**Fig. 13:**
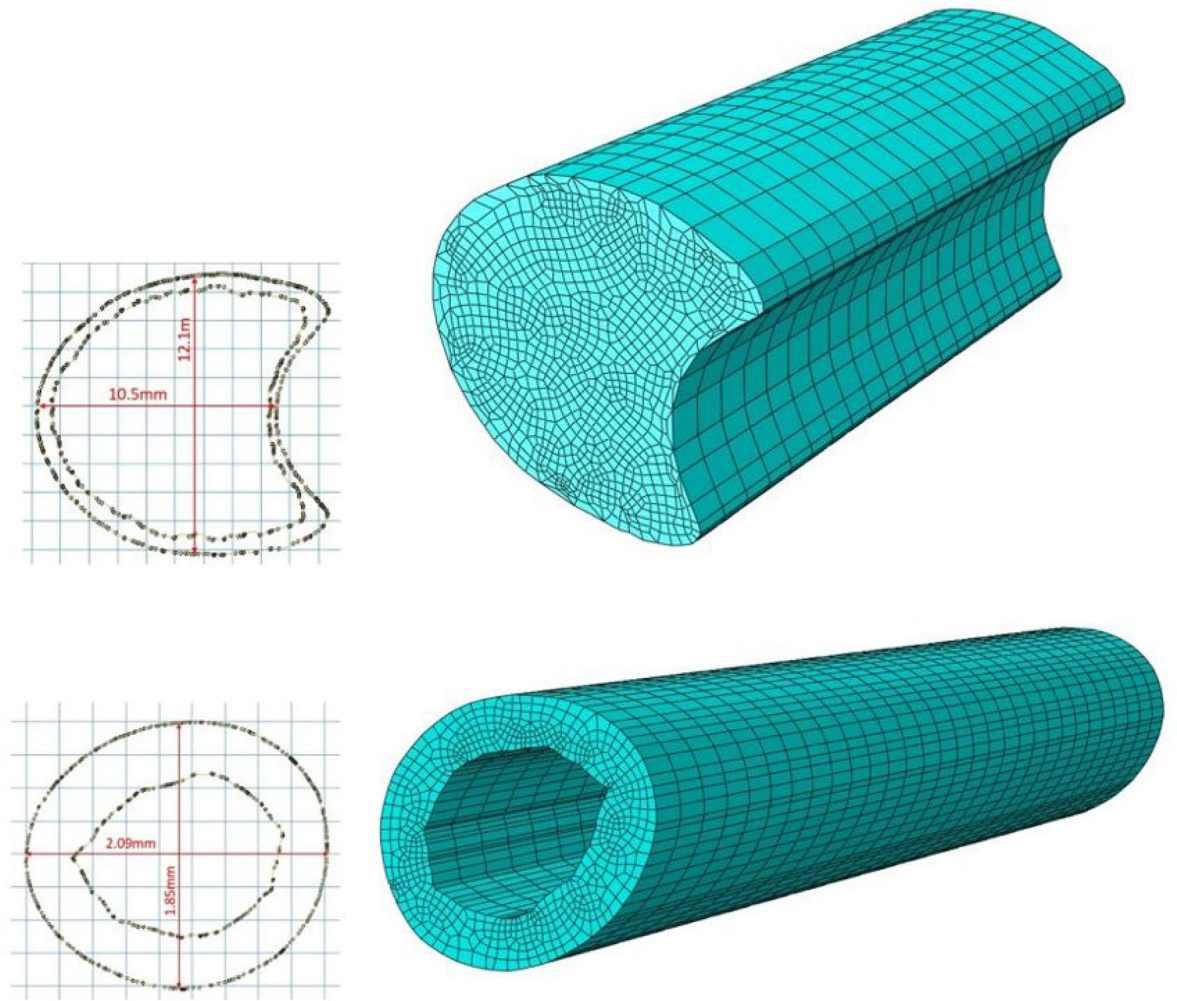
Undeformed three-dimensional model of maize (top) and wheat (bottom) internodes and their cross-sections

**Fig. 14:**
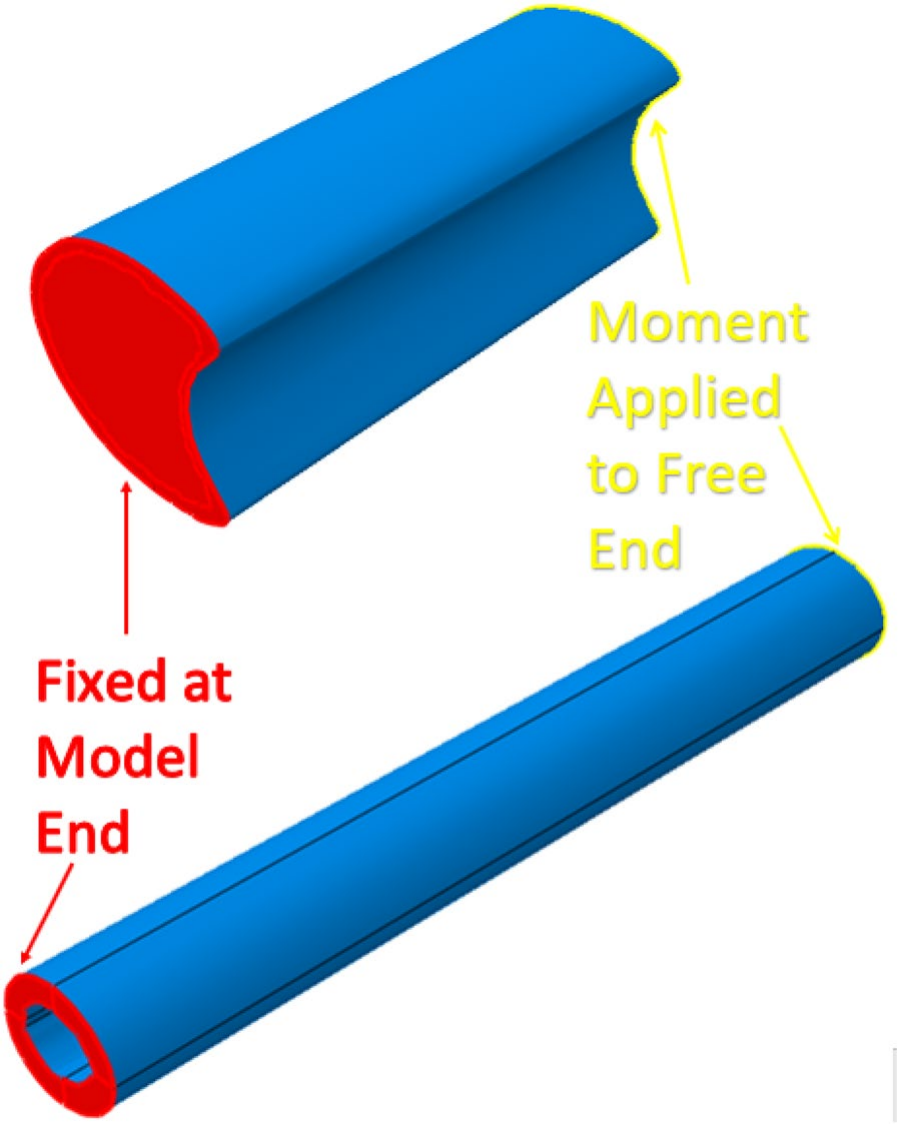
Boundary conditions and loads applied to maize (top) and wheat (bottom) stalk internode models

Several three-dimensional finite element models were also created and subjected to bending loads. In particular, specimen-specific geometries of maize and wheat samples were used to create three-dimensional models. The three-dimensional geometry of the samples was created using specimen-specific cross-sectional geometry that was then extruded in the z-direction. The models were meshed with eight-node linear three-dimensional stress brick reduced integration hexahedral elements as shown in Fig. 13. One cross-sectional surface of the model was fixed in all degrees of freedom while a bending moment was applied to the opposite cross-sectional surface in the minor axis direction (see Fig. 14). Low bending moments of 1Nm were applied to the maize and wheat model. Documented material properties of maize and wheat were assigned to the models. For maize a Young’s modulus of 4.41GPa and 20 MPa were assigned to the rind and pith respectively with a Poisson’s ratio of 0.2 [11]. For wheat a Young’s modulus of value of 2.23GPa was assigned to the rind with a Poisson’s ratio of 0.413 [45].

## Conclusion

A relative quick, simple, and inexpensive methodology has been developed to extract quantitative phenotypes from plant stalk cross-sections. A sample preparation methodology was presented wherein thin 1–4 mm sections of maize, poison hemlock, sorghum, wheat, and Arabidopsis were produced. These sections were stained using an Alcian Blue-Safranin O differential staining sequence. Quality digitized images of stained sections were obtained using a relatively inexpensive stereo microscope and 18MP camera. Digitized images were analyzed using an image processing algorithm developed in MATLAB to determine the major diameter, minor diameter, rind thickness and number of vascular bundles in each image. The MATLAB algorithm was also used to import the extracted phenotypes from each image into a third-party finite element software for further biomechanical analysis. Standard Operating Procedures and a description of each piece of equipment used in the study are provided as Additional files 1, 2 and 3 to enable other researchers to employ the developed phenotyping methodology.

## Supplementary Information

**Additional file 1.** Standard Operating Protocols.

**Additional file 2.** Matlab code.

**Additional file 3.** Instructions for Matlab code and sample images.

## Data Availability

All data has been uploaded as Additional files.
