# Supplementary material for: High throughput phenotyping of cross-sectional morphology to assess stalk lodging resistance
Source: Plant Methods. 2022 Jan 4;18:1. doi: 10.1186/s13007-021-00833-3 (PMC8725315; doi:10.1186/s13007-021-00833-3)
Supplement: Supplementary file 1 — Additional file 1. Standard Operating Protocols. [file 13007_2021_833_MOESM1_ESM.docx]

**Protocol for Phenotyping Maize, Sorghum or Poison Hemlock Stalk Cross-Sections**

*Sectioning*

- Identify stalks to section. Set the trim saw blade speed to a low setting of 2 and if required (for stiffer stalks) increase to 3 or 4. Hold internode segments firmly with saw vise and adjust saw fence according to required section thickness. Best thickness for staining sections is 2-4mm. Push the firmly held internode segments towards the rotating blade to cut sections.

*Staining*

- Preparation
  - Prepare stain solutions. Alcian Blue solution can be made by mixing 3grams of Alcian Blue, 250ml of distilled water, 6 drops of glacial acetic acid and 4ml of formaldehyde 40% solution. Safranin O Solution can be made by mixing 3grams of Safranin O, 100ml of Methylated Spirit, 100ml of distilled water and 2ml of formaldehyde 40% solution. Prepare stain solutions and keep for at least a day before use. Stain solutions can be stored and used for up to 6 months.
  - When staining sections, the staining solution and section to be stained are placed in a disposal cup. A second disposable cup with a hole punched in its bottom is then placed on top of the section to prevent it from floating up. Prepare all cups and staining solutions before starting the staining procedure.
- Staining Procedure
  - Hydrate sections in distilled water for 5 minutes.
  - Stain samples in Alcian Blue solution (a quick dip in the solution will typically suffice).
  - Rinse sections quickly in distilled water.
  - Stain samples in Safranin O (a quick dip in the solution will typically suffice).
  - Rinse sections quickly in distilled water.
  - Dehydrate sections in alcohol for 2 minutes.
  - Mount sections for imaging.

*Imaging*

- Prepare stereoscope and start Topview imaging software. In the Topview imaging software click on the camera that is connected to the stereoscope from ‘camera list’. In the Topview software select options to either save images in batches or to save a single individual image. Save all images in tagged image file (tif) format.
- Attach the 0.5X Barlow lens to stereoscope’s objective lens and set the stereoscope to an adequate height that ensures sharp, quality images can be captured. Calibrate the stereoscope according to the Amscope help manual (download help manual from camera software by clicking help contents in help tab). After calibration write down the calibration factor (number of millimeters per pixel) as it will be used as an input to the image processing algorithm for measurement conversion.
- Once calibrated AVOID MOVING CAMERA FROM SET POSITION. Any movement from its set position will require recalibration.
- Capture and save images of stained sections.

*Feature Extraction*

- Open image processing algorithm. For pith filled sections (i.e., maize and sorghum) open the “pith filled sections” MATLAB script. For hollow sections (i.e., poison hemlock) open the “hollow sections” MATLAB script. After opening the script assign input parameters (note descriptions of each input parameter are provided as comments in the MATLAB script).
- Run the MATLAB script. The MATLAB script will show the user an image of each section with overlaid vascular bundle boundaries as well as rind and pith boundaries. The user can then accept, reject or choose to manually select vascular bundles. The MATLAB script will then create a table which contains morphological measurements of the cross-section including major diameter, minor diameter, rind thickness, and number of vascular bundles. The MATLAB script will also create three Python script files that will be saved in the current working folder. These Python script files can subsequently be executed in Abacus to import sketches of the rind, pith, and vascular bundle boundaries.

*Geometry importation*

- Start Abaqus software and open a new file. Click on the file menu and select “Run Script”. Select one of the Python Script files that were created in the previous step.
- The selected file will be executed in Abaqus and a sketch of the stalk sample will be created. Repeat as needed to create sketches of the pith, rind, and vascular bundle boundaries.
- Follow standard model creation protocols to develop a finite element model based on the imported specimen specific geometries.

**Protocol for Phenotyping Wheat and Arabidopsis Stalk Cross-Sections**

*Sectioning*

- Identify stalks to section and prepare segments for cutting.
- Hydrate stalk segments in distilled water for 10 minutes.
- Remove stalk segments and hold the internode firmly in one hand with thumb and index finger and cut with other hand. Hold the blade perpendicular to internode and create sections simultaneously by chopping down in a single smooth motion. AVOID BACK AND FORTH SAWING MOTIONS. The best thickness for sections is 1-2mm.

*Staining*

- Follow the same staining procedure as outlined in the protocol for maize, sorghum, and poison hemlock.

*Imaging*

- Attach the 2.0X Barlow lens to the stereoscope and then follow the same imaging procedure as outline in the protocol for maize sorghum and poison hemlock.

*Feature Extraction*

- Follow the feature extraction procedure as outlined in the protocol for maize, sorghum, and poison hemlock.

*Geometry importation*

- Follow the geometry importation procedure as outlined in the protocol for maize, sorghum, and poison hemlock.
