# Supplementary material for: High throughput phenotyping of cross-sectional morphology to assess stalk lodging resistance
Source: Plant Methods. 2022 Jan 4;18:1. doi: 10.1186/s13007-021-00833-3 (PMC8725315; doi:10.1186/s13007-021-00833-3)
Supplement: Supplementary file 3 — Additional file 3. Instructions for Matlab code and sample images. [file 13007_2021_833_MOESM3_ESM.zip › How to Run Matlab Script.docx]

**How to Run MATLAB Script Used to Quantify Cross-sectional Phenotypes**

The MATLAB script requires two inputs from the user and outputs either four files (PithFilledSections.m) or three files (HollowSections.m) for each analyzed image.

**Inputs**

1. First, the script requires the folder path where the images to be analyzed are saved. The folder path is saved as a variable named (folderPathForInputImage). The path or address of the images to be analyzed should be inserted into the script on line 13 or line 14 in quotation marks (‘ ‘). For example, the provided MATLAB script has a folder path - ‘C:\Users\odun3492\Documents\microscopeImages’.
2. Second, the script requires a spatial calibration factor. The calibration factor is saved as a variable named (spatialCalibrationFactor). The standard operating protocols explain how to determine the spatial calibration factor for each image. The spatial calibration factor should be entered as mm/pixel. Spatial calibration factors for the sample images that were provided as supplementary data are shown in Table 1. Note that if you have images with different spatial calibration factors you will need to change the calibration factor in the MATLAB script and run the script on each image separately.

After inserting the required inputs, the user can run the MATLAB script. Dialogue boxes will pop-up to guide the user where required.

**Outputs**

The script will automatically generate an “Outputs” file folder. This file folder will be located in the same directory as the input image files. All outputs from the script will be saved in this folder.

1. The MATLAB script creates an excel file (PhenotypeMeasurements.xls). This file contains quantified phenotypes of cross-sectional images analyzed by the MATLAB script.
2. The MATLAB script creates several Python files. These files contain the rind, pith, vascular bundle, and, hollow area spline coordinates that can be used to import geometries into third part Abaqus software.
3. The MATLAB script creates an output image that shows the original input image with overlaid vascular bundles splines, as well as rind and pith boundaries.

**Table 1: Plant Stalk Cross-sectional Image and Associated Spatial Calibration Factor**

| **Image File** | **Spatial Calibration Factor** |
| --- | --- |
| Maize.tif | 1/164 (mm/pixel) |
| Sorghum.tif | 1/213 (mm/pixel) |
| PoisonHemlock.tif | 1/164 (mm/pixel) |
| Wheat.tif | 1/541 (mm/pixel) |
| Arabidopsis.tif | 1/10 (micron/pixel) |
