# Supplementary figures and images for: High throughput phenotyping of cross-sectional morphology to assess stalk lodging resistance
Source: Plant Methods. 2022 Jan 4;18:1. doi: 10.1186/s13007-021-00833-3 (PMC8725315; doi:10.1186/s13007-021-00833-3)

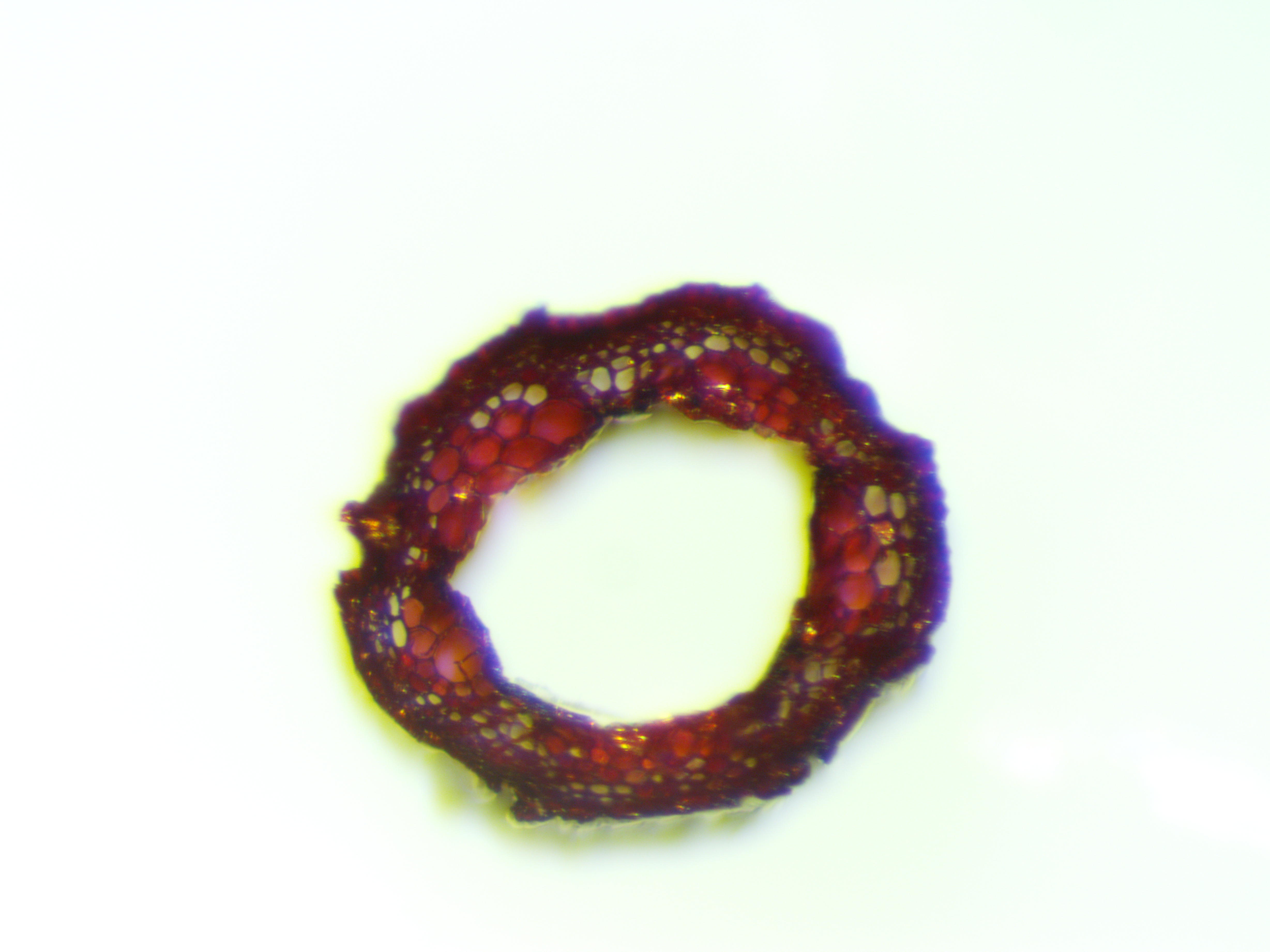

Supplement: Supplementary file 3 — Additional file 3. Instructions for Matlab code and sample images. [file 13007_2021_833_MOESM3_ESM.zip › Arabidopsis.tif]

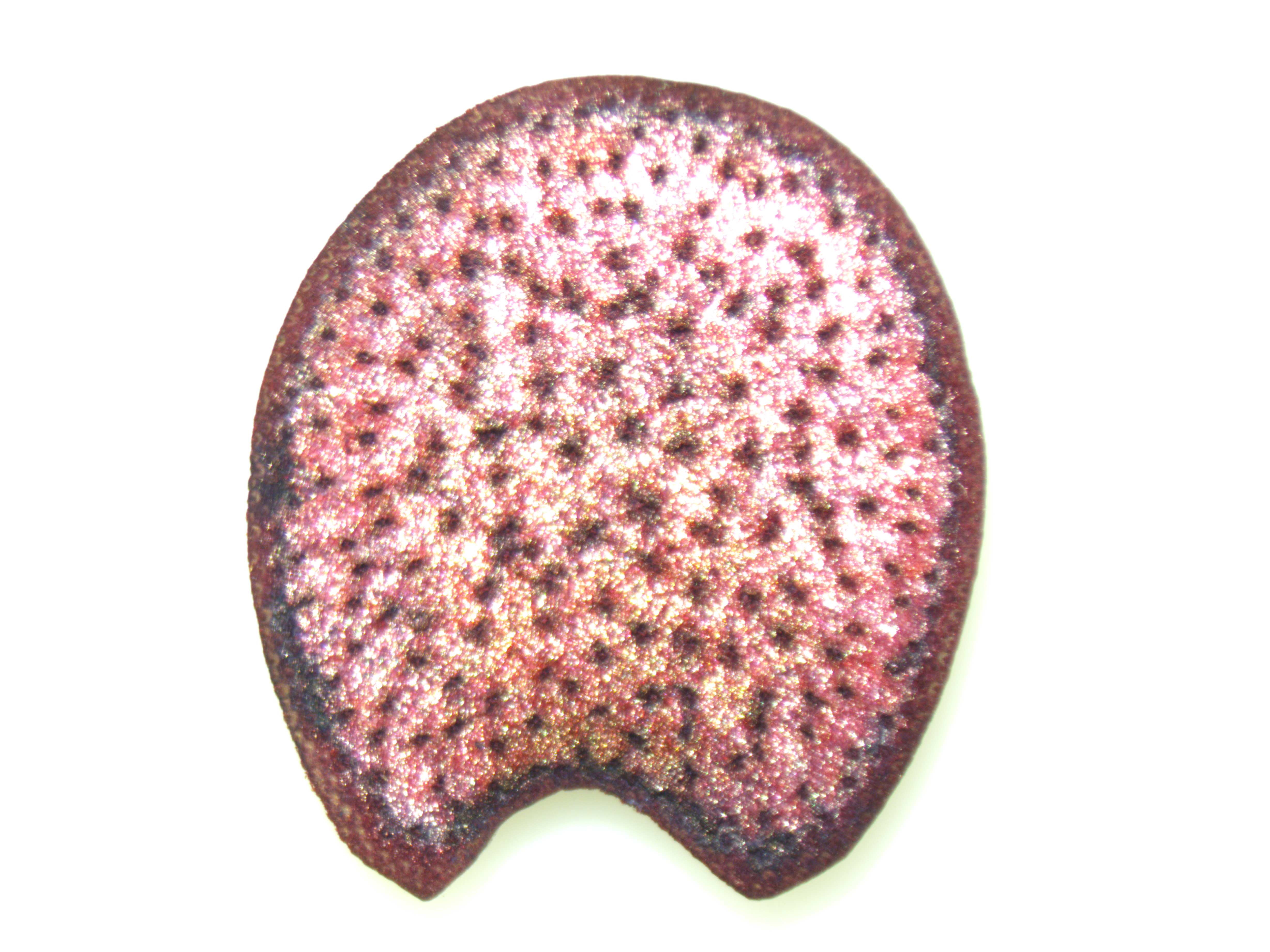

Supplement: Supplementary file 3 — Additional file 3. Instructions for Matlab code and sample images. [file 13007_2021_833_MOESM3_ESM.zip › Maize.tif]

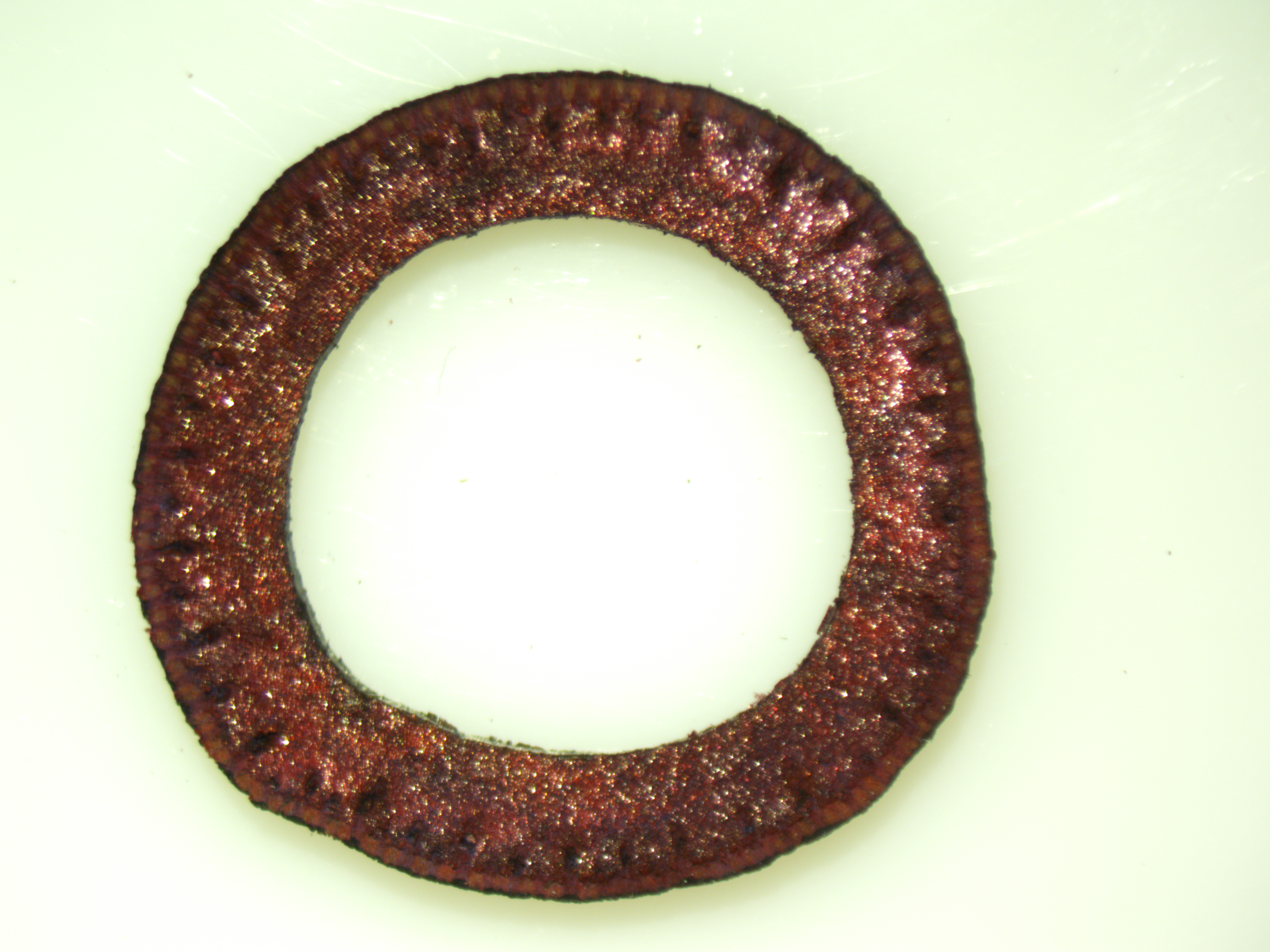

Supplement: Supplementary file 3 — Additional file 3. Instructions for Matlab code and sample images. [file 13007_2021_833_MOESM3_ESM.zip › PoisonHemlock.tif]

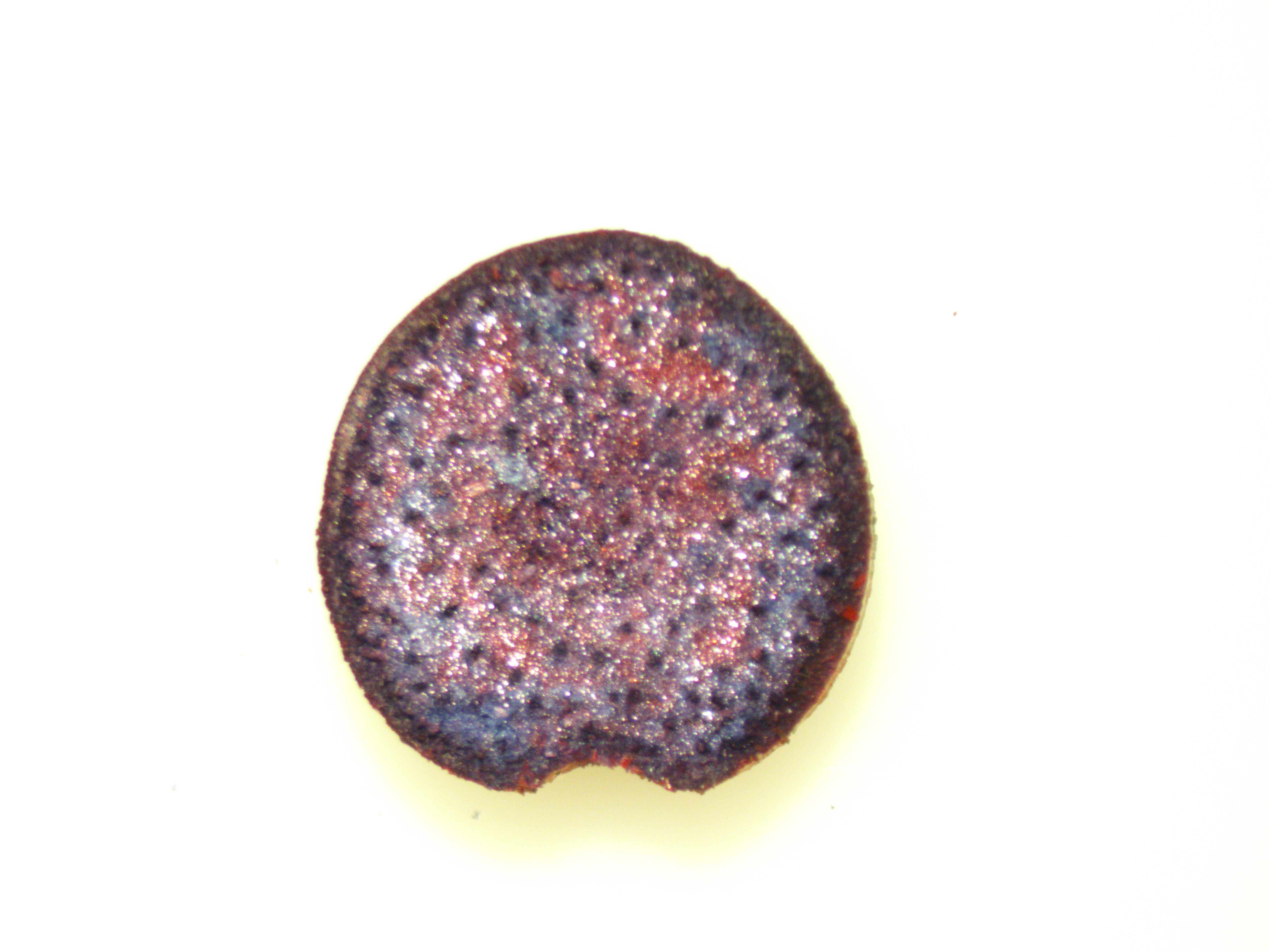

Supplement: Supplementary file 3 — Additional file 3. Instructions for Matlab code and sample images. [file 13007_2021_833_MOESM3_ESM.zip › Sorghum.tif]

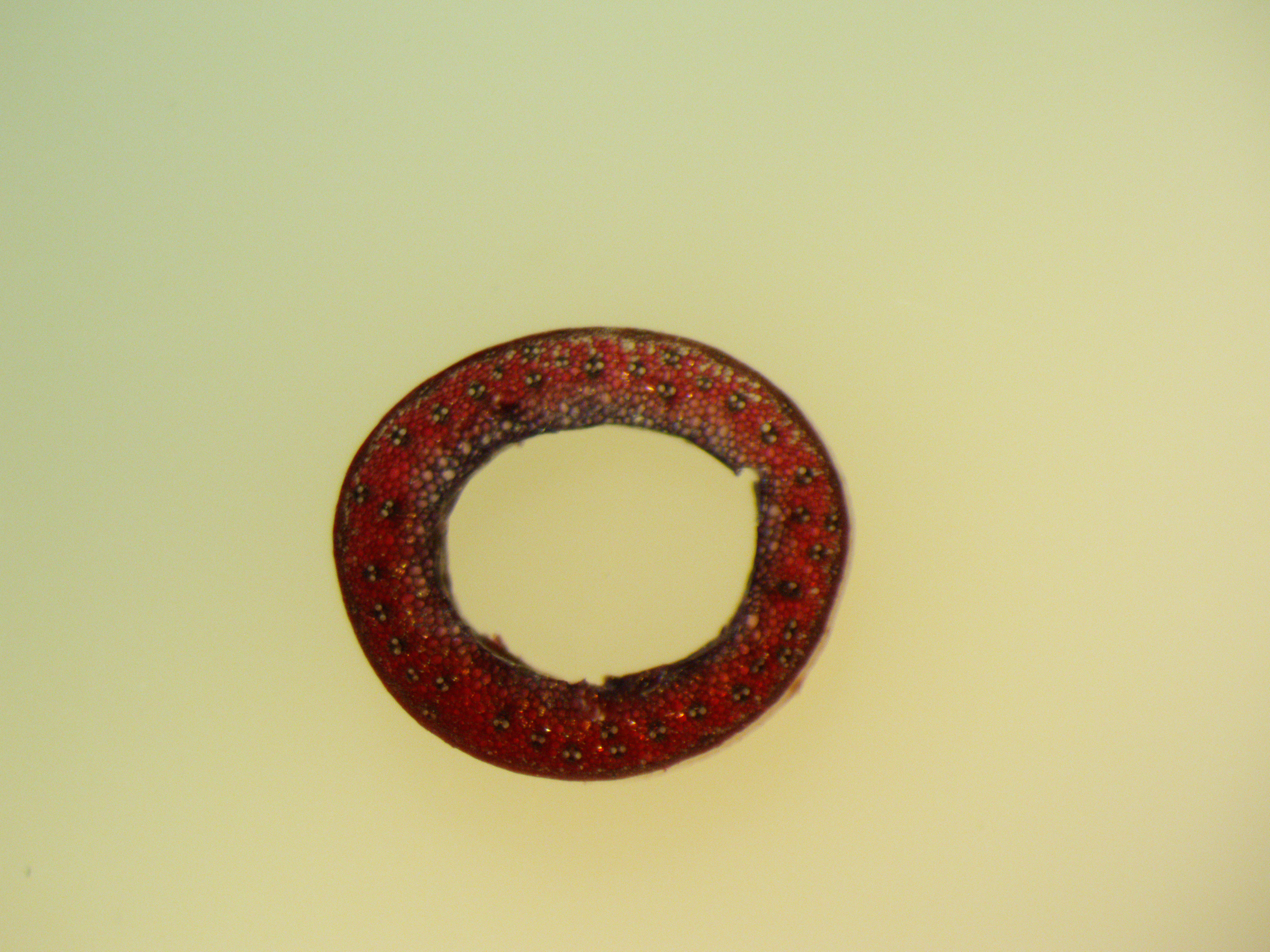

Supplement: Supplementary file 3 — Additional file 3. Instructions for Matlab code and sample images. [file 13007_2021_833_MOESM3_ESM.zip › Wheat.tif]
